# Anterior chamber proliferative membrane interception (AC-PMI)-enhanced trabeculectomy versus trabeculectomy for treating neovascular glaucoma: protocol for a randomized controlled trial

**DOI:** 10.1186/s13063-024-08123-8

**Published:** 2024-04-29

**Authors:** Shuqing Zhu, Mengtian Zhou, Haoyu Li, Shaodan Zhang, Shuxia Xu, Haishuang Lin, Yanqie Xie, Rongrong Le, Yuanbo Liang

**Affiliations:** 1 The Eye Hospital, School of Optometry and Ophthalmology, Wenzhou Medical University, Wenzhou, 325027 China; 2 Glaucoma Institute, Wenzhou Medical University, No.270 Xueyuanxi Rd, Wenzhou, 325027 China; 3 National Clinical Research Center for Ocular Diseases, Eye Hospital, Wenzhou Medical University, Wenzhou, 325027 China

**Keywords:** Ophthalmology, Neovascular glaucoma, Surgery

## Abstract

**Background:**

Neovascular glaucoma (NVG) is an irreversible blinding eye disease worldwide and is classified as one of the refractory glaucoma conditions, severely impacting visual function and vision. Unfortunately, effective surgical interventions to improve the prognosis of NVG patients are currently lacking. The study aims to evaluate the efficacy and safety of anterior chamber proliferative membrane interception (AC-PMI)-enhanced trabeculectomy compared to the traditional trabeculectomy.

**Methods:**

AC-PMI enhanced trabeculectomy versus trabeculectomy for the treatment of NVG is a single-center, prospective, double-arms, and randomized controlled trial of superior efficacy, which will involve 100 NVG inpatients. Patients will be randomly assigned into two groups using the random number table method. One group will undergo trabeculectomy using anti-vascular endothelial growth factor (Anti-VEGF) preoperatively and mitomycin C intraoperatively, while the other group will undergo AC-PMI enhanced trabeculectomy with the same medications (Anti-VEGF and mitomycin C). The patients will be followed up at the baseline and 1 day, 1 week, 1 month, 3 months, 6 months, 12 months, 18 months, and 24 months postoperatively. Meanwhile, we will collect the demographics, characteristics, and examination results and monitor any occurrences of adverse events at each follow-up time.

**Discussion:**

This is an efficacy study of a novel surgical approach for treating neovascular glaucoma. Building upon conventional filtering surgeries, this approach introduces an additional step involving the interception of the proliferative membrane to effectively halt the growth of fibrovascular tissue. This study aims to explore a promising new surgical approach for managing NVG and contribute to the advancement of glaucoma treatment strategies.

**Trial registration:**

ChiCTR ChiCTR2200055138. Registered on 01 January 2022. https://www.chictr.org.cn/showproj.html?proj=145255.

**Supplementary Information:**

The online version contains supplementary material available at 10.1186/s13063-024-08123-8.

## Introduction

Efforts to study effective prevention and treatment measures for neovascular glaucoma are essential. Glaucoma is a common optic neuropathy and stands as a leading cause of irreversible global blindness. As specific secondary glaucoma, neovascular glaucoma (NVG) tends to occur in patients with systemic disease. Clinically, NVG often arises as a consequence of conditions like diabetic retinopathy (DR), ischemic central retinal vein occlusion (CRVO), and ocular ischemic syndrome (OIS) [1]. The term “NVG” was introduced by Weiss in 1963, building on Coats’ earlier description of iris rubeosis in 1906 [2]. Nevertheless, it is difficult to make a breakthrough in the treatment of NVG, given the complexity of the primary disease involved and the unknown pathogenesis [3]. A typical characteristic of NVG is the neovascularization of the iris (NVI), which obstructs the chamber angle, leading to an increase in intraocular pressure (IOP). Current treatment approaches often involve the use of anti-vascular endothelial growth factor (Anti-VEGF) and fundus photocoagulation. However, despite these efforts, many patients continue to suffer from secondary glaucoma and may require additional medical or surgical interventions.

Currently, the surgical treatment of NVG still has disadvantages. The fundamental principle of the treatment of NVG is anting the neovascular and inflammation of the eye, managing the primary diseases, and lowering the IOP [4]. In clinical practice, it has been challenging to control the IOP of NVG patients by medication alone, which often requires surgical intervention. Filtration surgeries, such as trabeculectomy, drainage valve implantation, and ciliary body cryotherapy, are commonly employed. It has been found that NVG is the independent risk factor for the failure of filtration bleb-dependent surgeries [5]. Intraocular injection of Anti-VEGF can effectively control active pathological neovascularization, reduce the bleeding rate, and improve the success rate of surgery [6–8]. Additionally, the utilization of mitomycin C and other anti-metabolic agents during the operation has significantly improved the success rate [9, 10]. Drainage valve implantation is also an effective surgery for NVG, but its long-term success rate is not as optimistic [11, 12]. Studies have shown that the overall effect of trabeculectomy is equivalent to drainage valve implantation [7, 13, 14]. Although the success rate of a single operation in drainage valve implantation may be higher than that in trabeculectomy, drainage valve implantation has more long-term complications and a worse visual prognosis [15]. More high-quality studies are needed to demonstrate the different clinical effects of drainage valve implantation and trabeculectomy for NVG treatment [16]. In addition, the use of drainage valve implantation results in significantly increased costs. In this research, we aim to comprehensively consider the effect and patients’ acceptability of different surgeries, combining previous clinical practice and literature experience, with trabeculectomy chosen as the control.

Anterior chamber proliferative membrane interception (AC-PMI)-enhanced trabeculectomy shows promising potential for treating NVG patients. In response to the challenges posed by NVG, we have innovatively improved traditional surgery by integrating AC-PMI with trabeculectomy. During this procedure, we suture the cornea and iris in the corresponding region of the filter, creating an intercept membrane dike that ensures unobstructed filtration. This new technology, named Anterior Chamber Proliferative Membrane Interception (AC-PMI), effectively intercepts the neovascular membrane in the anterior segment. Notably, the half-year success rate of modified surgery based on trabeculectomy exceeded 85%.

The clinical effect and safety of AC-PMI enhanced trabeculectomy require further exploration. Although this modified operation is efficacy, its long-term efficacy and the difference between it and the traditional operation need to be proved by clinical trials. To address this, we have designed and conducted a clinical trial aimed at investigating the differences in efficacy between AC-PMI enhanced trabeculectomy and traditional trabeculectomy, confirming the efficacy and safety of this modified surgical approach.

## Methods

### Design overview

We take a superior effective trial to demonstrate AC-PMI enhanced trabeculectomy has a better clinical efficacy than traditional trabeculectomy and aim to provide a neoteric approach to NVG treatment. The trial involves nine follow-up times: baseline assessment before surgery, and postoperative follow-ups at 1 day, 1 week (± 3 days), 1 month (± 7 days), 3 months (± 7 days), 6 months (± 7 days), 12 months (± 7 days), 18 months (± 7 days), and 24 months (± 7 days). During these follow-up times, we will collect the information of patients. The first is demographics and characteristics, including the history of pretreatment, research measures, and the use of medicines. The second is intraocular pressure (IOP), visual acuity, slit-lamp examination, the outcome of perimetry, and corneal endothelial cell counting. The last is the occurrence of adverse events to evaluate the safety.

For each subject, the last visit will be considered the end of the trial for the individual. The completion of the last visit for all subjects in the trial will signify the conclusion of the entire trial.

### Sample size calculation

Based on the published results and previous studies, we assume that the average IOP after traditional trabeculectomy (using anti-VEGF drugs and mitomycin C) for NVG treatment is 17 mmHg; the average IOP after modified trabeculectomy (using anti-VEGF drugs and mitomycin C) is 12 mmHg. The pooled standard deviation is 3 mmHg, and we set the optimal boundary at 3.4 mmHg (representing 20% of the IOP change in the control group).

To estimate the required sample size, we consider a significance level (*α*) of 0.05, a statistical power (confidence, *1* − β) of 80%, and a ratio of 1:1 between the groups. Additionally, we consider an annual loss of follow-up rate of 10%. Based on these considerations, each group will require 50 patients, resulting in a total of 100 participants.

### Study subjects and grouping method

The staff of the Clinical Research Center presented the study to eligible patients at the glaucoma doctors’ outpatient clinics in the Eye Hospital, Wenzhou Medical University. Upon the staff or implementer obtaining the patient’s informed consent, AC-RMI combined with trabeculectomy was conducted, followed by regular follow-ups. Patients were provided with complimentary examinations at the Clinical Research Center of our hospital, and postoperative reviews were offered free of charge.

Patients diagnosed with neovascular glaucoma, receiving treatment at the Eye Hospital, Wenzhou Medical University, and willing to undergo AC-RMI enhanced trabeculectomy are eligible for inclusion. The specific inclusion and exclusion criteria are as follows:

The inclusion criteria for this study are as follows: (1) signed informed consent and willingness to follow up according to the specified time in the trial; (2) age 18–75 years, either sex; (3) diagnosed as neovascular glaucoma with neovascularization on iris and angle; (4) IOP remain greater than 21 mmHg (1 mmHg = 0.133 kPa) despite using the maximum IOP lowering drugs.

The exclusion criteria for this study are as follows: (1) inability to sign informed consent or unwillingness to participate in the follow-up or randomly enrolled; (2) suffered NVG secondary to intraocular tumors or inflammation; (3) presence of serious cardiovascular diseases or recent active inflammation in the eyes; (4) history of previous surgeries including cyclophotocoagulation, scleral encircling, trabeculectomy, drainage valve implantation, and silicone oil tamponade in the vitreous cavity; (5) female patients who are planning pregnancy, during pregnancy, or lactation; (6) patients with uncontrolled systemic diseases or judged unsuitable for this study due to other reasons.

The staff of the Clinical Research Center who is not involved in assessing the outcome of the study uses software to generate a random distribution sequence for determining patient grouping. The grouping results are then placed into sequentially numbered, sealed, opaque envelopes. When a patient meets the criteria, the implementer opens the envelope with the corresponding number to confirm the group of the patient. As the study is an invented surgery study, the implementer and surgeon are not blinded; only the outcome evaluator and patients are blinded. Patients will be informed that they will undergo trabeculectomy, but they do not know whether the AC-PMI will be used.

If emergencies or severe adverse events occur, the outcome evaluator or the subject of the grouping will be informed. Once the blindness is broken, the subject will be regarded as a lost subject and their data may be excluded from the analysis.

### Interventions

In order to eliminate neovascularization of the iris to reduce intraoperative bleeding, both groups of patients received an intravitreal injection of anti-VEGF approximately 1 week before the operation. Intervention will be provided by Dr Liang, an experienced glaucoma physician.

Control group (undergo traditional trabeculectomy) procedure: Surgeries were performed after local subconjunctival anesthesia, and the superior rectus muscle was suspended. The conjunctiva was incised along the corneal limbus at the 11:00–12:00–1:00 o’clock positions. A 4.0 mm × 3.0 mm scleral flap was created with the base at the 12:00 o’clock position, extending approximately 1 mm into the clear cornea. A cotton slice soaked with 0.3 mg/ml mitomycin C was placed under the scleral flap for 4 min, followed by thorough irrigation with a large amount of saline. Trabecular tissue measuring about 1.0 mm × 1.5 mm was excised, along with the peripheral iris measuring about 1.5 mm × 2.0 mm. The scleral flap was then sutured with 2 interrupted sutures of 10-0 polypropylene. Physiological saline was injected into the anterior chamber through the side incision to confirm the functioning of the scleral flap’s filtration. The scleral flap was sutured with interrupted 10-0 sutures. After completion of the procedure, the conjunctival sac was covered with a combination of neomycin and dexamethasone eye ointment.

**Fig. 1:**
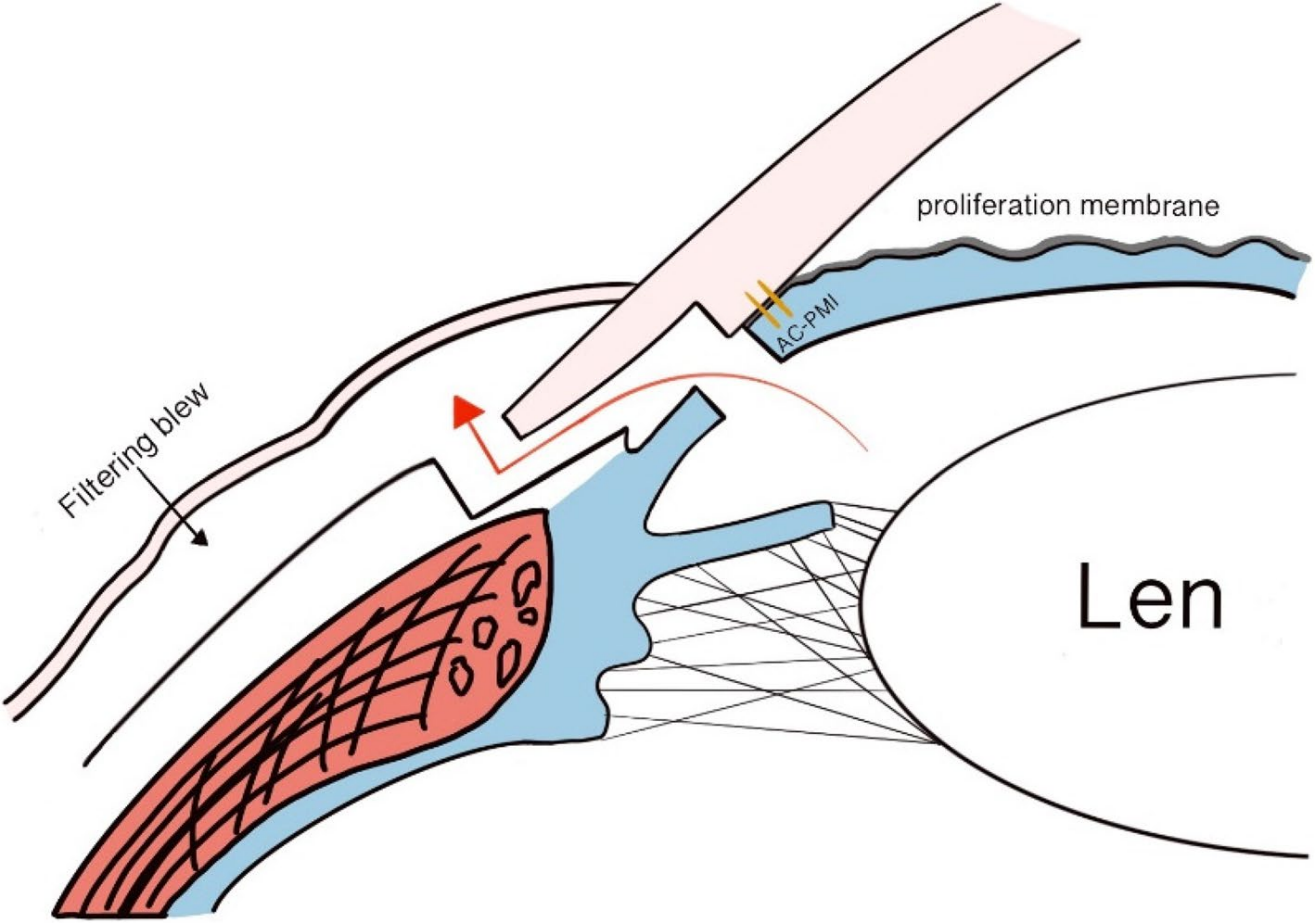
Surgical diagram of the experimental group. The gray lines represent the fibrovascular membrane proliferating on the anterior surface of the iris. The two orange straight lines represent the artificially sutured interception dams (AC-PMI). The red arrow indicates the aqueous humor outflow pathway

Experimental group (undergo AC-PMI-enhanced trabeculectomy): Based on traditional trabeculectomy, this method sutures the cornea and iris before excising the trabecular tissue and the peripheral iris located behind the intercepted portion (Fig. 1).

The specific surgical procedure for the experimental group is as follows: Similarly, under local subconjunctival anesthesia with suspension of the superior rectus muscle, an incision was made along the corneal limbus at the 11:00-1:00 o’clock positions to create a conjunctival flap. A 4.0 mm × 3.0 mm scleral flap with the base at the 12:00 o’clock position was made, extending to expose the clear cornea. A cotton pad soaked with 0.3 mg/ml mitomycin C was placed under the scleral flap for 4 min, followed by extensive irrigation with saline. A corneal side incision was made at the 9:00 o’clock position, and viscoelastic (Healon GV) was injected into the posterior chamber through the pupil area to appose the peripheral iris to the corneal endothelium. At the filtration site corresponding to 12:00 o’clock, 10-0 polypropylene sutures were used to intermittently suture 2-3 stitches, approximately 2.0 mm away from the corneoscleral limbus, with a width of about 3.5 mm × 4.0 mm, securing the iris to the corneal edge. The tissue behind the sutures was excised, including a trabecular meshwork and peripheral iris tissue of the same size as the control group. Finally, 10-0 polypropylene sutures were used to intermittently suture the scleral flap with 2 stitches. Physiological saline was injected through the side incision to form the anterior chamber, confirming the good filtration function at the scleral flap site. The conjunctival flap was then intermittently sutured with 10-0 polypropylene sutures. At the end of the procedure, neomycin and dexamethasone eye ointment were applied to the patient’s conjunctival sac.

### Study process

We treat and follow up for 24 months with the patients, 9 times in total. 100 patients who meet the inclusion criteria will be assigned into two groups randomly. Subsequently, the patients will be followed up for a period of 24 months, with a total of 9 follow-up visits at specific time points: post-operation 1 day, 1 week (7 ± 3 days), 1 month (30 ± 7 days), 3 months (90 ± 7 days), 6 months (180 ± 7 days), 12 months (360 ± 7 days), 18 months (540 ± 7 days), 24 months (720 ± 7 days). Educational guidance on follow-up times will be provided to participants at the time of their agreement to participate. Additionally, 1 week before each follow-up appointment, patients will be contacted by telephone to remind them and schedule their visit to our hospital for reexamination.

**Fig. 2:**
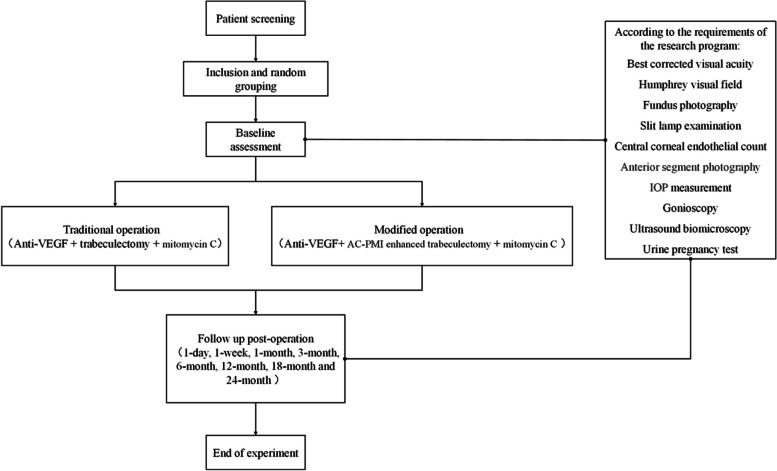
Flow chart of the study

During these follow-up visits, relevant inspections will be conducted, and efficacy and safety assessments will be performed. Additionally, the case report form (CRF) will be completed for data collection and analysis. The examination plan of each follow-up time is shown in Table 1, and the flow chart is shown in Fig. 2.

**Table 1:** The examination plan of each following-up time

Follow-up time	Preoperation	Post-operation							
	Baseline	1 day	1 week	1 month	3 months	6 months	12 months	18 months	24 months
Informed consent	×								
Demography	×								
Medical history collection	×								
Inclusion/exclusion criteria	×								
Urine pregnancy test	×			×	×	×	×	×	
Slit lamp examination	×	×	×	×	×	×	×	×	×
Gonioscopy examination	×		×	×	×	×	×	×	×
Best-corrected visual acuity	×		×	×	×	×	×	×	×
IOP measurement	×	×	×	×	×	×	×	×	×
Anterior segment photography	×		×	×	×	×	×	×	×
Ultrasound biomicroscopy	×		×	×	×	×	×	×	×
Humphrey visual field	×				×	×	×	×	×
Corneal endothelial count	×		×	×	×	×	×	×	×
Fundus photography	×				×	×	×	×	×
Drug combination	×	×	×	×	×	×	×	×	×
Adverse events	×	×	×	×	×	×	×	×	×
Reoperation		×	×	×	×	×	×	×	×
End of the trial									×

Patients may exit from this study if the following occurs: (1) the patient actively requires to exit this study; (2) pregnant; (3) the patients need to stop treatment due to safety reasons (such as adverse events); (4) the patient who is necessary to combine other local or systemic drug therapy (such as hormones, immunosuppressive agents), physical therapy or surgical treatment due to the need for pathogenetic condition that has an impact on the condition of neovascular glaucoma; (5) participating in other clinical study or researchers thought patients needed to exit from the study for other reasons. If a patient lost to follow-up, researchers should contact the patient to determine the reason for stopping/exiting. We record the reasons for exiting the study in the CRF and the original file. If adverse events occur in patients, including ocular local adverse events and systemic adverse events, they should be recorded in CRF and corresponding measures should be taken.

If there are specific surgical complications related to AC-PMI enhanced trabeculectomy, such as corneal endothelial decompensation, we will correspondingly reduce or waive the patient’s treatment expenses.

### Main outcome measures

In this study, two main categories of outcome measures have been established: safety measures and efficacy measures. Safety measures include the following: (1) Corneal Endothelial Cell Count: The corneal endothelial cell count will be assessed at each follow-up visit and compared to the baseline measurement; (2) Incidence of Complications: The occurrence of complications related to the surgical procedures or treatments will be recorded at each follow-up time point; (3) Intergroup Difference in Safety Measures: The safety outcomes between the two treatment groups (Anti-VEGF therapy combined with AC-PMI-enhanced trabeculectomy vs. trabeculectomy alone) will be compared to identify any significant differences in terms of corneal endothelial cell count and complication rates. Efficacy measures include the following: (1) IOP; (2) the best-corrected visual acuity; (3) Intergroup Difference in Efficacy Measures. The IOP and the best-corrected visual acuity at every follow-up time compared to the baseline. In addition to the safety and efficacy measures, the study will also collect and analyze the following data: (1) Demographics: Patient characteristics such as age, gender, and relevant medical history will be collected to understand the population’s baseline characteristics. (2) Clinical characteristics: Specific clinical characteristics related to NVG, such as the neovascularization of the iris (NVI) or the neovascularization of the angle (NVA), and baseline ocular findings, will be recorded. (3) Other clinical inspections: Additional clinical assessments may be conducted to monitor the overall health of the patients and evaluate the impact of treatments on other ocular parameters.

According to the above indicators, the primary outcome measures include evaluating the changes in IOP and best-corrected visual acuity (BCVA) at each visit during the first-year post-treatment compared to the baseline. Additionally, the study aims to assess the differences in IOP and BCVA between the two treatment groups at each visit within the initial year post-treatment. We will statistically adjust for multiple comparisons using appropriate methods such as Bonferroni correction or other techniques for multiplicity adjustment. This approach will ensure clarity and robustness in our analysis. The secondary outcome measures involve analyzing the alterations in IOP and BCVA at each visit during the second year post-treatment compared to baseline. Furthermore, the study intends to examine the differences in IOP and BCVA between the two groups at each visit within the second year post-treatment.

We will adhere to the principles of complete and timely publication of research results. The study findings will be disseminated through publications such as papers and research reports, ensuring timely sharing with participants, healthcare professionals, and relevant communities.

### Ethics and data administration

The ethical committee approved this study protocol of the Eye Hospital, Wenzhou Medical University, in April 2023 (No: 2021-228-K-199-04). The written informed consent from the patients is required by the ethics committee. The main risk to participants may be adverse events occurring and a potential leak of privacy. As soon as any safety information appears in the study, the investigator should fill out the relevant CRF. Serious adverse events should be reported to the Research Center and the Ethics Committee within 24 h after being notified, describing the duration, severity, frequency, specific treatment, and outcome of the adverse events.

The medical records and relevant examination data from each center are directly uploaded to the electronic data management system of the National Eye Disease Clinical Medical Research Center through a centralized data transmission platform. All collected data are anonymized and do not contain any patient privacy information. Any content that could potentially identify the subject has been carefully removed. The data is securely managed by a dedicated data manager on the platform, and any request for data usage requires proper submission and approval.

The frequency of submission of data security and monitoring reports to the Ethics Committee is 1 per year. When the number of enrolled cases reaches half of the sample size and completes the follow-up at 6 months postoperatively, independent statisticians, unaware of the grouping, will conduct the primary outcome analysis. Paired *t*-tests or Wilcoxon signed-rank tests will be utilized to compare efficacy between the two groups. The results will be submitted to the Ethics Committee, and if *P* > 0.05, the Ethics Committee will deliberate on the potential termination of the trial due to futility.

### Statistical analyses

We will report the statistical results to assess the efficacy and safety of AC-PMI-enhanced trabeculectomy, and the difference with trabeculectomy in NVG patients. The continuous variables with normal distribution will be expressed as mean ± standard deviation, and variables not conforming to normal distribution will be expressed as median values (interquartile ranges). The discontinuous variables will be expressed as a percentage (%). All data will be tested by a two-sided test. The continuous variables with a normal distribution will be compared using a two-sample *t*-test, and a two-sided Mann–Whitney *U*-test will be used for that with a non-normal distribution. The values will be statistically significant at *P* < 0.05. Subgroup analysis will be performed based on age, sex, etiology, and history of PRP surgery. For efficacy data from a certain follow-up that cannot be utilized or is missing, if the subsequent follow-up data is valid and can be used, the efficacy data from the subsequent follow-up can be carried forward and used as the data for the current follow-up. We will handle protocol non-adherence statistically by employing an intention-to-treat (ITT) analysis approach. This method helps maintain the integrity of randomization and avoids biases that may arise from excluding non-adherent participants from the analysis. Additionally, we will conduct sensitivity analyses to assess the robustness of our findings and evaluate the impact of protocol non-adherence on the study outcomes.

## Discussion

With the diet structure changing, the incidence of diabetes is gradually increasing. According to IDF Diabetes Atlas (2021), the global incidence of diabetes in adults aged 20–79 has risen to 10.5%, which was 9.26% in 2019 and 8.4% in 2017. Diabetes is a significant risk factor for various complications, including diabetic retinopathy (DR). In patients with neovascular glaucoma (NVG), DR is reported to be the most common cause of the condition [17]. Consequently, the incidence of NVG is increasing in parallel with the rise in diabetes cases. Presently, the common surgeries for NVG such as trabeculectomy and drainage valve implantation are filter bubble dependent. For patients with advanced NVG who are difficult to control IOP with unbearable eye pain, cyclodestructive surgery is often used to decrease IOP. Nevertheless, the risk of ocular atrophy was high in this treatment. NVG patients are often accompanied by systemic diseases, poor compliance, and severe visual impairment, which further complicate the management of their condition.

The pathogenesis of NVG is complex, which is secondary to fundus ischemia, hypoxia, and neovascularization even the fibrovascular membranes formed in the anterior segment of the eye. As for recurrent neovascularization, the success rate of glaucoma surgery decreased. We use the AC-PMI technology combined with traditional trabeculectomy to block the crawling of fibrovascular membranes and produce this study to find the efficacy of this surgery.

The AC-PMI technique aims to intercept the recurrent fibrovascular membrane, thereby preventing the contraction and blockage of the filtration outlet caused by the appearance of the fibrovascular membrane. This, in turn, helps avoid surgical failure. We have conducted a retrospective study of the effect of AC-PMI-enhanced trabeculectomy in NVG patients showed promising results, with a complete success rate of 72.6% and a conditional success rate of 87.3% at 12 months after the operation [18]. However, that were only 33 eyes included, and no control group, we design this randomized controlled trial to further study its efficacy. At the same time, we hope this neoteric surgery may provide a novel treatment option for NVG.

The study acknowledges its limitations, including the potential influence of the patient’s systemic disease control on the treatment effect. Blood pressure, blood glucose levels, and carotid artery imaging examinations were not collected at each follow-up visit. Additionally, the chosen surgery, trabeculectomy combined with AC-PMI, is similar to other external filtering glaucoma surgeries, necessitating filter bubble maintenance in the later stages. If AC-PMI proves effective for NVG patients, the researchers propose combining it with non-filtering bleb-dependent surgery to address the challenge of filtering bleb scarring.

Overall, this study aims to explore a promising new surgical approach for managing NVG and contribute to the advancement of glaucoma treatment strategies.

## Trial status

The version number of this research protocol is Version 2.1, 28 April 2022. The recruitment began date is 11 April 2022, and the date when recruitment will be completed is 31 December 2023.

Amendments to the research protocol, including the modified study protocol and informed consent, with specific dates and version numbers, will be submitted to the Ethics Committee. Simultaneously, the updated study information will be posted on the Chinese Clinical Trial Registry website. After approval from the Ethics Committee and verification on the registry website, the modified protocol will be implemented.

### Supplementary Information

**Additional file 1.** Informed Consent. Contact Information.

## Data Availability

The data underlying this article cannot be shared publicly due to the privacy of individuals who participated in the study. The data will be shared on reasonable request to the corresponding author.
