# Supplementary material for: Anterior chamber proliferative membrane interception (AC-PMI)-enhanced trabeculectomy versus trabeculectomy for treating neovascular glaucoma: protocol for a randomized controlled trial
Source: Trials. 2024 Apr 29;25:287. doi: 10.1186/s13063-024-08123-8 (PMC11057066; doi:10.1186/s13063-024-08123-8)
Supplement: Supplementary file 1 — Additional file 1. Informed Consent. Contact Information. [file 13063_2024_8123_MOESM1_ESM.docx]

# Informed Consent

**Experimental title:** Randomized Controlled Trial for Anterior Chamber Proliferative Membrane Interception (AC-PMI)-enhanced Trabeculectomy Versus Trabeculectomy for the Treatment Neovascular Glaucoma

**Research institute:** Glaucoma Institute, Wenzhou Medical University

We would like to invite you to participate in a clinical trial titled "Randomized Controlled Trial for AC-PMI-enhanced Trabeculectomy Versus Trabeculectomy for the Treatment Neovascular Glaucoma". Your specific circumstances meet the inclusion criteria for this study, and therefore, we would like to extend this invitation to you. This informed consent form will introduce you to the purpose, procedures, benefits, risks, inconveniences, or discomforts associated with this study. Please read it carefully before making a considered decision on whether to participate. During the discussion of this informed consent form with the research staff, you are welcome to ask questions and have any unclear points explained to you. You can also discuss this decision with your family, friends, and your primary healthcare provider before making a decision.

1.Why is this trial being conducted?

Glaucoma is the leading cause of irreversible blindness worldwide, with various subtypes, including neovascular glaucoma, which is a challenging secondary form. Currently, the primary treatment for neovascular glaucoma is trabeculectomy (combined with anti-angiogenic and anti-fibrotic agents), showing significant clinical outcomes. Building upon this, our team has innovatively introduced the Anterior Chamber Proliferative Membrane Interception (AC-PMI) technique, an enhanced version of trabeculectomy incorporating an interception step to control fibrovascular tissue growth. This clinical trial aims to systematically investigate the clinical efficacy of this modified procedure.

2.What does this trial involve?

This study involves observing patients with neovascular glaucoma who have undergone either the modified or conventional trabeculectomy. We will assess factors such as intraocular pressure, visual acuity and corneal endothelial cell counts to evaluate the effectiveness and safety of the AC-PMI technique in neovascular glaucoma treatment.

1. How many participants will be involved in this trial?

A total of 100 patients with neovascular glaucoma will participate in this clinical trial, divided into two groups of 50 individuals each. Participants will be randomly assigned to either receive traditional or modified trabeculectomy.

4.Who is eligible to participate in the trial?

Participants who meet the following criteria may be eligible:

1. Willingness to sign informed consent and adhere to follow-up requirements.
2. Age between 18 and 75, regardless of gender.
3. Diagnosis of neovascular glaucoma with neovascularization on the iris and angle.
4. Intraocular pressure remaining above 21 mmHg despite maximum use of IOP-lowering drugs.

5.Who cannot participate in the trial?

Individuals who meet any of the following criteria will be excluded:

1. Inability to sign informed consent or unwillingness to participate or follow-up.
2. Neovascular glaucoma secondary to intraocular tumors or inflammation.
3. Presence of severe cardiovascular diseases or recent ocular inflammation.
4. History of eye surgeries such as cyclodestructive procedures, scleral encircling, drainage valve implantation, or silicone oil tamponade.
5. Pregnant, planning pregnancy, or currently breastfeeding.
6. Uncontrolled systemic diseases or other reasons deemed unsuitable by the researchers.
7. Who might withdraw from the trial?

Participants may withdraw from the trial if any of the following occur:

1. Voluntary request to exit the trial.
2. Pregnancy.
3. Necessity to cease treatment due to safety concerns (such as adverse events).
4. Requirement for combined therapy with other local or systemic medications or interventions.
5. Participation in other clinical trials or researcher's decision to withdraw the patient for other reasons.
6. Study Methods

This is a 24-month randomized controlled trial, involving 9 visits: baseline and visits at postoperative day 1, 1 week, 1 month, 3 months, 6 months, 12 months, 18 months, and 24 months. Eligible patients will be randomly assigned to receive either conventional or modified trabeculectomy. During the study, basic patient information, medical history, and medication use will be collected. Efficacy assessments, including best-corrected visual acuity, intraocular pressure measurements, slit-lamp examinations, visual field tests, and corneal endothelial cell counts, will be conducted at each visit. Adverse events occurring during the study will be documented for safety assessment.

1. Risks of the Study

Risks associated with the study may include anesthesia-related accidents, drug allergies, cardiovascular incidents, changes in surgical plans due to evolving conditions, postoperative infections, delayed wound healing, complications such as retinal detachment, corneal decompensation, inflammation, decreased postoperative visual acuity, intraoperative bleeding requiring hemostatic measures, unsatisfactory intraocular pressure control necessitating further interventions, and choroidal detachment.

1. Benefits of the Study

Participants will directly benefit from this trial as both the modified and traditional trabeculectomy procedures have the potential to alleviate or cure neovascular glaucoma by controlling intraocular pressure, thus enhancing their quality of life. Participation offers a waiver of registration fees for six months and free ophthalmic examinations related to the study.

1. Alternative Treatment Options

If participants choose not to take part in this study, alternative treatment options include procedures such as transscleral cyclophotocoagulation and drainage valve implantation.

1. Your Rights

Participants have the right to decide whether to join the trial. If immediate decision-making is not possible, participants have ample time for consideration. Trusted individuals like family members or friends can be consulted before a decision is made. Opting out of the trial will not impact the relationship with the researchers, and participants will not face discrimination or reprisal.

1. Travel and Medical Compensation

This study does not offer travel reimbursement. In the event of complications directly linked to the AC-PMI technique (e.g., corneal decompensation), corresponding treatment expenses will be waived.

1. Privacy Protection

Personal information provided to researchers (such as name, gender, contact details, survey responses) may be accessed by personnel associated with the research funding institution (monitors, auditors) and administrative bodies like national and local food and drug supervision authorities. However, without consent, no individual or entity other than researchers and administrative authorities has the right to disclose personal information or directly contact participants regarding the trial.

1. Additional Considerations
2. Participants may be withdrawn from the study without their consent for the sake of their health if any of the following situations occur:

- Risks outweigh benefits.
- Participant does not adhere to the study protocol.
- Premature termination of the trial.

1. This informed consent document consists of two copies—one for the participant and one for the researcher.
2. Contact Information
3. Ethics Committee Office of Wenzhou Medical University Affiliated Eye and Vision Hospital

Contact: 0577-88075582

(2)Principal Investigator of the Study—Yuanbo Liang

Contact: 0577-88053506

**Contact Information**

1. I have thoroughly read the Participant Information and am aware of the trial's background. The researcher has provided detailed explanations of the study's characteristics, potential adverse reactions, and answered my questions.
2. I understand that declining participation in this trial will not affect my treatment and rights. After considering the entire Participant Information and reflecting upon it, I voluntarily choose to participate in this trial.
3. I am willing to follow the researcher's instructions and participate in the trial as per the study protocol. During the trial, I have the right to withdraw at any time, provided I notify the researcher promptly.
4. If any discomfort arises during the trial, I will promptly inform the researcher.

Participant Signature:

|  | Year/ Month/ day |
| --- | --- |
| Signature: | Signature Date |

Researcher Signature:

|  | Year/ Month/ day |
| --- | --- |
| Signature: | Signature Date |

Participant's Representative/Guardian (if applicable):

Reason for Participant's Inability to Sign:

Relationship between Representative/Guardian and Participant:

|  | Year/ Month/ day |
| --- | --- |
| Signature: | Signature Date |
